# Does pregabalin offer potential as a first-line therapy for generalized anxiety disorder? A meta-analysis of efficacy, safety, and cost-effectiveness

**DOI:** 10.3389/fphar.2025.1483770

**Published:** 2025-02-07

**Authors:** Narcis Cardoner, Luis Gutiérrez-Rojas, Pilar Saiz, Guillermo Lahera, Miguel Ángel Álvarez-Mon, Pino Alonso Ortega, María Pérez-Páramo

**Affiliations:** ^1^ Psychiatry Department, Hospital de la Santa Creu i Sant Pau, Barcelona, Spain; ^2^ Institut d’Investigació Biomèdica Sant Pau IIB SANT PAU, Hospital de la Santa Creu i Sant Pau, Barcelona, Spain; ^3^ Centro de Investigación Biomédica En Red en Salud Mental CIBERSAM, Instituto de Salud Carlos III, Madrid, Spain; ^4^ Department of Psychiatry and Forensic Medicine, School of Medicine Bellaterra, Universitat Autònoma de Barcelona, Barcelona, Spain; ^5^ Department of Psychiatry and CTS-549 Research Group, Institute of Neurosciences, University of Granada, Granada, Spain; ^6^ Department of Psychiatry, University of Granada, Granada, Spain; ^7^ Department of Psychiatry, University of Oviedo, Oviedo, Spain; ^8^ CIBER de Salud Mental, Instituto de Salud Carlos III, Madrid, Spain; ^9^ Instituto de Investigación Sanitaria del Principado de Asturias (ISPA), Oviedo, Spain; ^10^ Instituto Universitario de Neurociencias del Principado de Asturias (INEUROPA), Oviedo, Spain; ^11^ Servicio de Salud del Principado de Asturias (SESPA) Oviedo, Oviedo, Spain; ^12^ Department of Psychiatry, Universidad de Alcalá, Madrid, Spain; ^13^ Príncipe de Asturias University Hospital, Oviedo, Spain; ^14^ Instituto de Investigación Sanitaria Ramón y Cajal (IRyCIS), Madrid, Spain; ^15^ Department of Psychiatry and Mental Health, Hospital Universitario Infanta Leonor, Madrid, Spain; ^16^ Department of Medicine and Medical Specialties, University of Alcala, Alcala de Henares, Spain; ^17^ Ramón y Cajal Institute of Sanitary Research (IRYCIS), Madrid, Spain; ^18^ Department of Psychiatry, Hospital de Bellvitge, Barcelona, Spain; ^19^ Bellvitge Biomedical Research Institute (IDIBELL), Barcelona, Spain; ^20^ Department of Clinical Sciences, University of Barcelona, Barcelona, Spain; ^21^ Medical Department, Viatris, Madrid, Spain

**Keywords:** pregabalin, benzodiazepines, SSRIs, SNRIs generalized anxiety disorder, efficacy, safety

## Abstract

**Introduction:**

Generalized Anxiety Disorder (GAD) is a mental health condition with a recent increase in prevalence. GAD is often underdiagnosed, leading to negative consequences for individuals, healthcare systems, and society. The economic burden and impaired quality of life associated with GAD underscores the need for effective treatment. Pregabalin has shown promise in reducing anxiety symptoms; however, further research is needed to evaluate its efficacy and compare it with other treatment options. This study aimed to assess the efficacy, safety, and optimal pregabalin dosage for the treatment of GAD.

**Methods:**

This meta-analysis followed PRISMA guidelines. Pregabalin-treated patients comprised the intervention group, whereas the comparator group received benzodiazepines, SSRIs, SNRIs, or placebo. Efficacy and safety were evaluated using various scales and adverse events (AEs). Randomized clinical trials were included in the study. Four major databases were used for this study. Outcome measures included the Hamilton Anxiety Rating Scale (HAM-A), Clinical Global Impression Improvement Scale (CGI-I), discontinuation rates, costs, and quality-adjusted life-years (QALYs). Meta-analyses were conducted using Review Manager 5.4 software, employing odds ratios (ORs) and mean differences (MDs) with 95% confidence intervals (CIs). Subgroup and sensitivity analyses were performed based on follow-up and dosage.

**Results:**

Fourteen studies involving 4,822 patients were analyzed. Pregabalin demonstrated superior efficacy in reducing HAM-A global scores at 2 weeks (MD −1.23, 95% CI −1.79 to −0.66), 4 weeks (MD −1.12, 95% CI −1.60 to −0.63), 8 weeks (MD −2.50, 95% CI −4.21 to −0.79), 12 weeks (MD 0.99, 95% CI 0.35–1.63), and 6 months to 1 year (MD −3.31, 95% CI −4.30 to −2.31). Pregabalin also showed a higher response rate to HAM-A (OR 1.51, 95% CI 1.31 1.75). CGI-I scores favored pregabalin (MD −0.25, 95% CI −0.38 to −0.12), with a higher response rate (OR 1.33, 95% CI 1.15–1.55). The discontinuation rates were lower with pregabalin (OR 0.80, 95% CI 0.70, 0.91). Adverse events favored pregabalin over SSRIs/SNRIs and benzodiazepines at different doses. Pregabalin was associated with higher cost-effectiveness (MD 0.02, 95% CI 0.01, 0.03).

**Conclusion:**

Pregabalin is an effective and well-tolerated treatment for generalized anxiety disorder, showing superior efficacy and safety compared with first-line medications.

**Systematic Review Registration:**

PROSPERO CRD42024556152.

## 1 Introduction

Generalized Anxiety Disorder (GAD) is a mental health condition with an estimated lifetime prevalence ranging from 8% to 13% (Haller et al., 2014; Armbrecht et al., 2021). Although after the COVID-19 pandemic the prevalence of anxiety disorders has risen to 25.6% (COVID-19 Mental Disorders Collaborators, 2021). Despite its high prevalence, Generalized Anxiety Disorder (GAD) is often underdiagnosed, leading to significant negative consequences for affected individuals, healthcare systems, and society (Haller et al., 2014; Armbrecht et al., 2021). The impact of GAD extends beyond the disorder itself, as it is associated with reduced work productivity, decreased quality of life, and an increased risk of comorbidity with other mental health disorders such as Major Depressive Disorder (MDD) or borderline personality disorder (BPD) (Armbrecht et al., 2021; Qadeer Shah et al., 2023).

A study by Revicki et al. (2012) found that the average annual cost of managing GAD in primary care exceeds 1,000 euros per patient compared to those without the disorder. This highlights the substantial economic burden associated with GAD, and the importance of early diagnosis and effective treatment. Furthermore, a systematic review by Bereza et al. (2009) revealed that individuals with GAD experience significant impairment in their quality of life. The study found that the quality of life reported by GAD patients is comparable to that of individuals 20 years old who are affected by somatic diseases such as Parkinson’s disease or heart failure. Another important aspect to highlight is the recurrent nature of GAD is its recurrent course. Studies have reported a recurrence rate of 24% at 2 years, with 22% partial recurrence and 30% complete recurrence (Scholten et al., 2013; Rodriguez et al., 2006). This, coupled with the significant burden associated with anxiety disorders, particularly GAD, emphasizes the need to continue exploring new, effective, and safe therapeutic alternatives for both short- and long-term management.

Several therapeutic options are currently available for the treatment of GAD. Benzodiazepines are widely and routinely prescribed, although not all are suitable for GAD. However, their long-term use presents risks and drawbacks, such as adverse cognitive effects, tolerance and dependence, and an increased risk of falls in the elderly (Paulose-Ram et al., 2004). As a result, selective serotonin and norepinephrine reuptake inhibitors (SNRIs) and selective serotonin reuptake inhibitors (SSRIs) have been established as first-line treatments because of their efficacy and favorable side effect profiles (DeMartini et al., 2019). SSRIs approved for GAD include paroxetine and escitalopram, whereas SNRIs approved for GAD are venlafaxine and duloxetine. Pregabalin, another first-line drug, acts by binding to the α2δ subunit of voltage-dependent calcium channels in the central nervous system, reducing the release of neurotransmitters involved in various processes, such as seizure, pain signaling, and the regulation of emotional responses (Alles et al., 2020). The α2δ subunit is an auxiliary subunit of voltage-gated calcium channels, and pregabalin modulation leads to decreased neuronal excitability (Scholten et al., 2013). This mechanism of action is believed to underlie the anticonvulsant, analgesic, and anxiolytic effects of pregabalin (Scholten et al., 2013). In addition to its effects on calcium channels, pregabalin has been shown to modulates norepinephrine and substance P levels, which may contribute to its therapeutic properties (Alles et al., 2020). The mechanism of action of pregabalin in the treatment of GAD is considered beneficial because of its ability to modulate neurotransmitter release and reduce the excessive neuronal activity associated with anxiety (Alles et al., 2020). Pregabalin has been approved for the treatment of GAD.

The efficacy of pregabalin in treating Generalized Anxiety Disorder (GAD) has been extensively investigated in individual studies and meta-analyses. Individual studies have shown a significant reduction in both the psychological and somatic symptoms of anxiety (Feltner et al., 2008; Montgomery et al., 2008). Moreover, from an economic management perspective, pregabalin has been found to be a cost-effective alternative to other treatments such as venlafaxine (Silva Miguel et al., 2013). A meta-analysis conducted by Boschen (2011) found pregabalin to be an effective treatment for GAD with a moderate effect size in reducing overall anxiety symptoms. This study revealed that pregabalin had a moderate effect on both psychic and somatic anxiety symptoms, indicating its ability to address the psychological and physical manifestations of anxiety. In a comparative meta-analysis, Generoso et al. (2017) evaluated the efficacy of pregabalin in the treatment of GAD. The results showed comparable clinical response rates between the two treatments. Interestingly, pregabalin demonstrated lower dropout rates than benzodiazepines, suggesting better tolerability and fewer side effects associated with pregabalin. A systematic review and meta-analysis by Kong et al. (2020) investigated the use of pregabalin in various anxiety spectrum disorders. This study found a consistent effect favoring gabapentinoids, including pregabalin, over placebo. Moreover, the tolerability of pregabalin was comparable to that of placebo, indicating that it was relatively well tolerated among the studied drugs.

However, these meta-analyses have significant limitations that preclude the drawing of definitive conclusions. First, they included a heterogeneous mix of anxiety disorders, such as social anxiety disorder, rather than focusing solely on pure GAD. Additionally, most comparisons are limited to placebo rather than other active treatment options, such as SSRIs, SNRIs, or benzodiazepines. There are also shortcomings in the analysis and control of data heterogeneity as well as a lack of statistical pooling of data. Another notable issue is the absence of subgroup analysis according to variables, such as follow-up time, dose, or control drug used in the studies. These limitations underscore the need for more rigorous and comprehensive research to adequately assess the efficacy and compare different treatment options for GAD.

This study aimed to evaluate the efficacy and safety of pregabalin compared with placebo and other well-established treatment alternatives, such as SSRIs, SNRIs, and benzodiazepines, for the treatment of GAD. Furthermore, different doses of pregabalin were compared to optimize their efficacy in the treatment of GAD.

## 2 Materials and methods

### 2.1 Eligibility criteria

**FIGURE 1:**
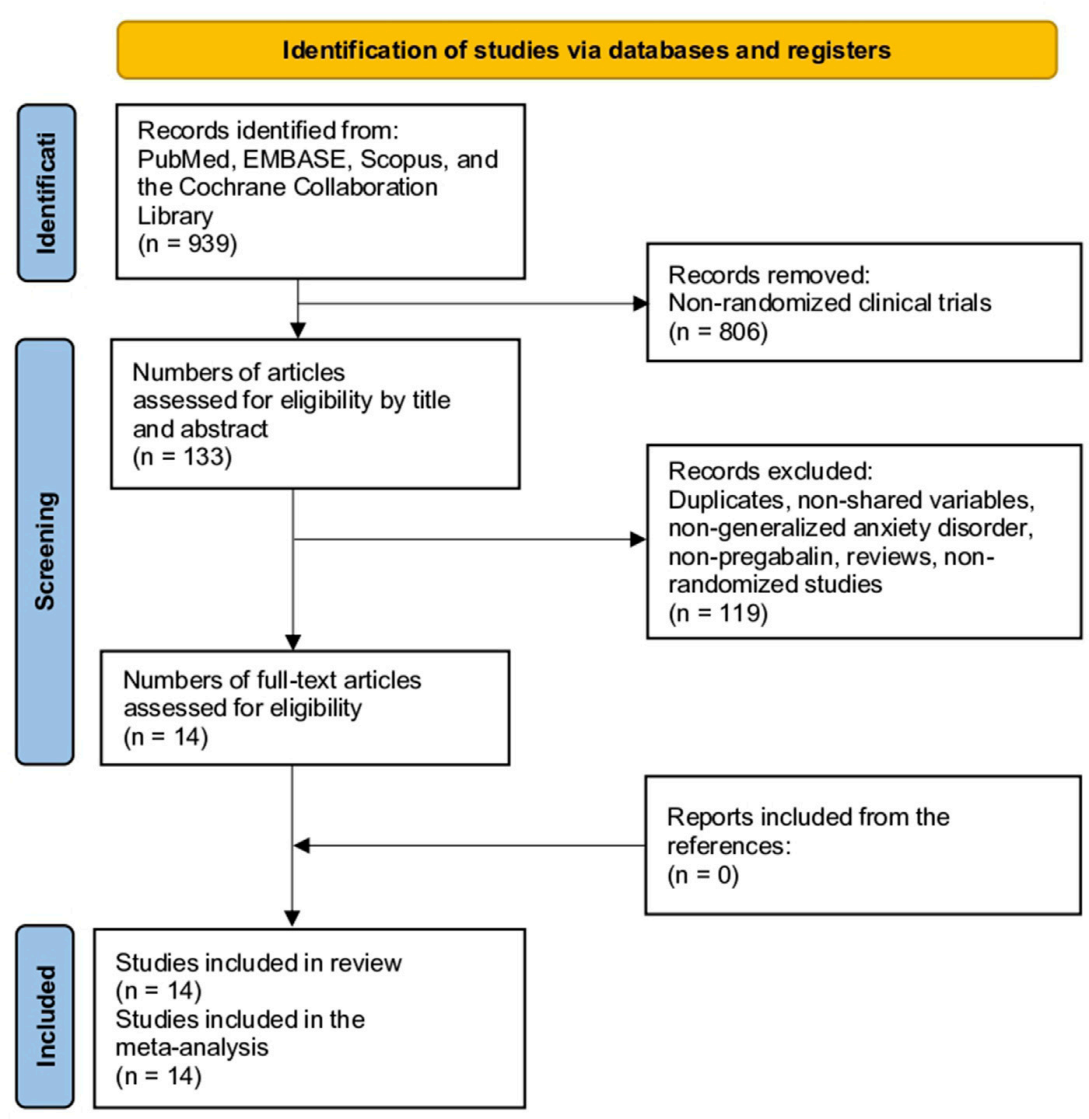
Study selection flow diagram (Preferred Reporting Items for Systematic reviews and meta-analyses).

This meta-analysis followed the protocol registered in the International Prospective Register of Systematic Reviews (PROSPERO) and adhered to the Preferred Reporting Items for Systematic Reviews and Meta-Analyses (PRISMA) guidelines (Page et al., 2021) (Figure 1). The PICOS (patients, intervention, comparison, outcomes, and study design) strategy was used to select the studies: P) adult patients diagnosed with GAD. The inclusion criteria were clear and required patients to meet the DSM-IV criteria for GAD using a structured interview, such as the Mini-International Neuropsychiatric Interview (MINI) (Sheehan et al., 1998). On the other hand, exclusion criteria were established to exclude patients with other axis I disorders, except for dysthymia, specific phobia, social phobia, somatization disorder, or a history of major depressive disorder. Patients at risk of suicide, with current severity of suicidal ideation, those with a current diagnosis of seizure disorder, or a history of bipolar disorder, schizophrenia, psychotic disorder, or factitious disorder were also excluded. Patients in the intervention group (I) were treated with pregabalin, while those in the comparator group (C) were treated with benzodiazepines, SSRIs, SNRIs, or placebo. The outcomes of interest (O) were efficacy and safety, evaluated using different scales, and adverse events, respectively. The included studies (S) were randomized clinical trials.

To ensure the quality and coherence of this study, rigorous exclusion criteria were applied. First, non-randomized studies, such as case reports, case series, editorials, cohort studies, case-control studies, cross-sectional studies, and protocols, were excluded. This decision was made to ensure that the data obtained were from randomized clinical trials, which are considered the gold standard in scientific research. Additionally, pediatric populations were excluded, as the focus of this study was on adult patients. Duplicate studies were excluded to avoid data duplication and ensure the integrity of the analysis. In the case of duplicate studies reporting related information in different publications, the decision was made to group them into a single study to avoid duplication of basic characteristics and to ensure that each variable was accounted for only once. However, relevant information was extracted from each duplicate study for inclusion in the analysis. This allowed the utilization of complementary data and ensured a comprehensive evaluation of the available results without incurring unnecessary redundancy. Studies with incomplete data or those in which variables were not comparable were also discarded, as this could have affected the validity and reliability of the results.

### 2.2 Information sources

Comprehensive searches for information were conducted across multiple sources, including PubMed, EMBASE, SCOPUS, and the Cochrane Library, with no restrictions on date or language. Additionally, a manual search of the reference lists from the included studies was performed, which allowed for the identification of additional studies that might have been missed in the electronic searches.

### 2.3 Search methods for identification of studies

The search strategy used in this study targeted the keywords “pregabalin” or “Lyrica” and “anxiety” (Supplementary Material 1). Two reviewers independently screened the titles and abstracts of the selected studies. Any discrepancies or disagreements between reviewers were resolved through discussion with a third reviewer.

### 2.4 Data extraction and data items

Two reviewers conducted the screening process, and any disagreements were resolved by consensus with a third reviewer. Baseline characteristic variables extracted from the included studies included study details, region, period, follow-up duration, sample size, age, female participants, duration of GAD, doses of pregabalin or control, conflict of interest (COI), and funding sources. Additional information was obtained regarding the treatment schemes and drug-free periods.

The primary outcome measures were the Hamilton Anxiety Rating Scale (HAM-A) score and adverse events. The HAM-A is a widely used clinical tool for measuring the severity of anxiety symptoms in patients, ranging from 0 to 56, with higher scores indicating greater anxiety severity (Thompson, 2015). It was also the responder rate according to the HAM-A scale, defined as a 50% or greater reduction from baseline in the HAM-A total score or a HAM-A score of 7 or less. The subdomains of the HAM-A scale, namely, psychic and somatic, were also compared. The Clinical Global Impression Improvement Scale (CGI-I) was also extracted, which measures the overall change in a patient’s clinical condition using a 7-point scale, with 1 indicating “very much improved” and 7 indicating “very much worse” (Busner and Targum, 2007). The responder rate to the CGI-I was defined as a CGI-I score of 2 or less (much or very much improved). Furthermore, the number of patients discontinuing treatment in each group, reasons for discontinuation (lack of efficacy or adverse events), costs of the drug, total costs, and quality-adjusted life years (QALYs) were evaluated. Sleep disturbances were assessed using the HAM-A (insomnia subdomain), and the Medical Outcomes Study Sleep Scale (MOS-Sleep) score was also evaluated. It incorporates 12 items covering different aspects of sleep quality and sleep disturbances. These items assess sleep disturbances, sleep adequacy, sleepiness, sleep quantity, snoring, and awakening with shortness of breath or headache (Viala-Danten et al., 2008). Finally, the adverse events were compared.

### 2.5 Assessment of risk of bias in included studies

**FIGURE 2:**
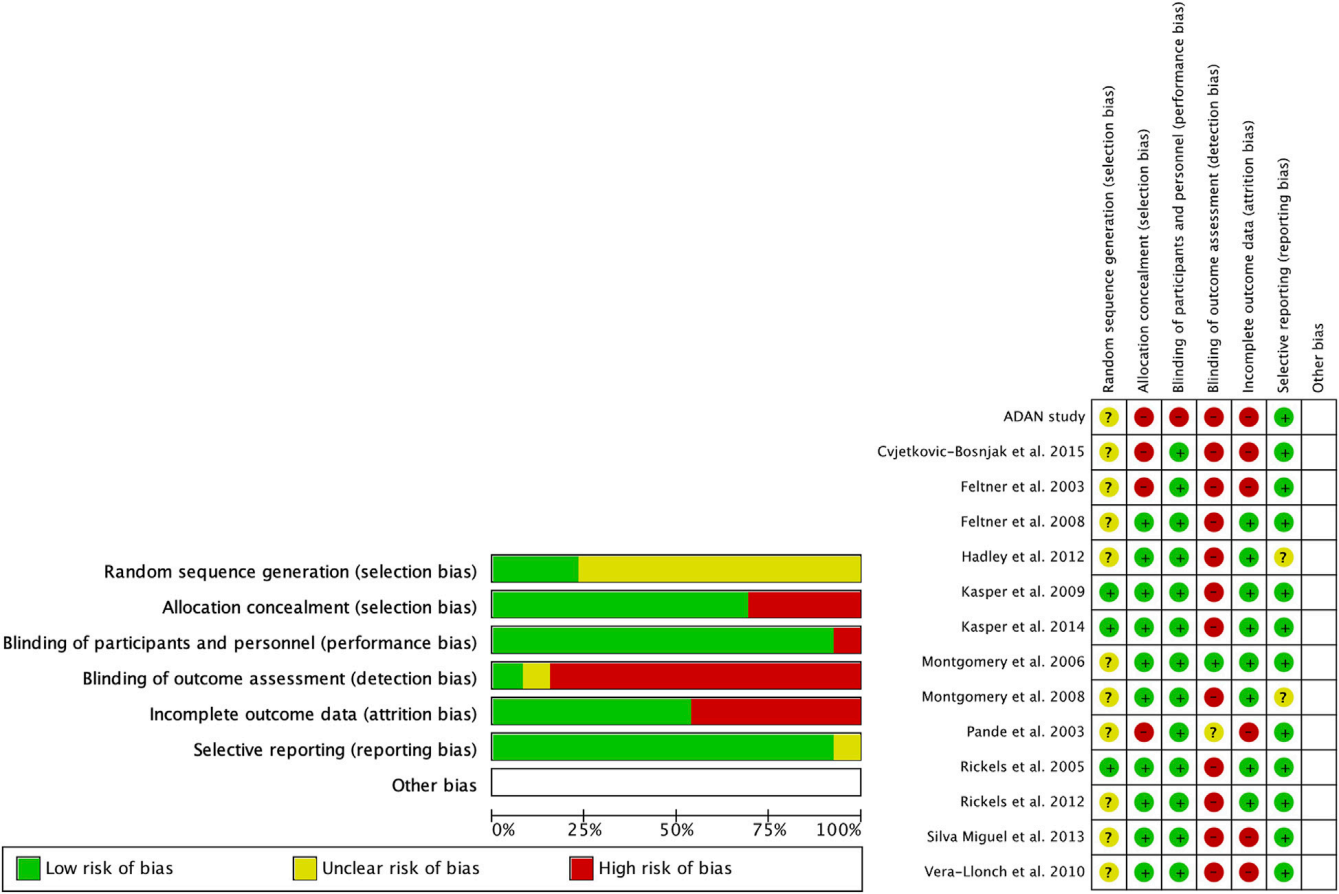
Risk of bias (green = low risk; red = high risk; yellow = unknown).

The risk of bias assessment was conducted by two reviewers using the Cochrane Collaboration tool for randomized controlled trials in the Review Manager 5.4 software. The following six domains were considered: random sequence generation, allocation concealment, blinding of participants and personnel, blinding of outcome assessments, incomplete outcome data, and selective reporting. Each domain was evaluated and assigned a judgment of a low, high, or unclear risk of bias. The results of the risk of bias assessment are shown in Figure 2; Supplementary Material 2. Additionally, forest plots displayed the risk of bias grading for each trial, providing a comprehensive overview of the potential biases in the included studies.

### 2.6 Assessment of results

Meta-analyses were conducted using Review Manager 5.4 software. Odds ratios (ORs) with corresponding 95% confidence intervals (CIs) were calculated for dichotomous variables. Continuous variables were analyzed using the mean differences (MDs) or standardized mean differences (SMDs) with 95% CIs, depending on whether the studies had compatible units or scales. Heterogeneity was assessed using the chi-square statistic and I2 index, where I2 values of 25%, 50%, and 75% represented low, moderate, and high levels of heterogeneity, respectively. A fixed-effects model was used in cases of homogeneity, whereas a random-effects model was applied in the presence of heterogeneity. Precise data points from the study figures were extracted using the WebPlotDigitizer software version 13.1.4. Missing data were addressed following the guidelines outlined in the Cochrane Handbook to ensure a comprehensive analysis (Higgins et al., 2019).

### 2.7 Risk of bias across the studies

Publication bias was evaluated using funnel plots that were visually generated using Review Manager version 5.4. Funnel plots depict the effect estimates of the analyzed studies on the *x*-axis, while the standard errors are represented on the *y*-axis. These plots provide a graphical representation of potential publication bias in the included studies. In an ideal scenario without publication bias, the plot would display a symmetrical funnel shape, indicating that smaller studies with larger standard errors are scattered more widely around the estimated effect size, whereas larger studies with smaller standard errors are clustered closely to the estimated effect size. However, asymmetry in the funnel plot may suggest the presence of publication or other sources of bias. It is important to note that no formal statistical tests were conducted in this assessment of publication bias, and the interpretation was based on visual inspection of funnel plots.

### 2.8 Additional analyses

Subgroup analyses were conducted based on the duration of follow-up, categorizing outcomes into short-term (2 and 4 weeks), medium-term (8 and 12 weeks), and long-term (6 months–1 year) outcomes. Subgroup analyses were performed according to dosage. Owing to heterogeneity and variation in the dosage employed across studies, the dosage was divided into low-dose (less than 300 mg/day) and high-dose (greater than 300 mg/day) pregabalin. If studies provided different dosages, the results were reported separately for each dosage type rather than in combination.

Sensitivity analyses were conducted to explore the effects of separate comparisons: pregabalin vs. placebo, pregabalin vs. benzodiazepine, pregabalin vs. selective serotonin reuptake inhibitors (SSRIs), and pregabalin vs. serotonin-noradrenaline reuptake inhibitors (SNRIs).

The Grading of Recommendations Assessment, Development, and Evaluation (GRADE) approach utilizing GRADEpro was employed to assess the certainty of the outcomes (Guyatt et al., 2013). GRADE evaluates the quality of evidence based on factors such as the study design, risk of bias, inconsistency, indirectness, imprecision, and publication bias.

## 3 Results

### 3.1 Study selection

The initial search yielded 939 studies. After reviewing the titles and abstracts and excluding non-randomized studies, 806 studies were eliminated, resulting in 133 articles. After excluding studies that did not compare pregabalin with placebo, other drugs for the indication of GAD, or other types of reviews, 99 studies were eliminated, leaving 34 studies. After reviewing the full text, 20 studies were eliminated because of duplication or not being used for GAD, resulting in 14 studies. After examining all references in these studies, no additional studies were added (Figure 1) (Feltner et al., 2008; Montgomery et al., 2008; Silva Miguel et al., 2013; Rickels et al., 2005; Feltner et al., 2003; Kasper et al., 2014; Pande et al., 2003; Hadley et al., 2012; Rickels et al., 2012; Álvarez et al., 2015; Cvjetkovic-Bosnjak et al., 2015; Kasper et al., 2009; Vera-Llonch et al., 2010; Montgomery et al., 2006).

### 3.2 Risk of bias

Regarding the risk of bias (Figure 2), the studies were randomized; however, the mode of randomization was not reported in most cases. The evaluators of the results were not blinded, and in approximately half of the cases, patients who did not reach the end of the follow-up were not reported.

### 3.3 Study characteristics

Table 1 presents the basic characteristics of the studies included. Fourteen studies and 4,822 patients were included (2,650 in the pregabalin group, 432 in the benzodiazepine group, 792 in the SSRI/SNRI group, and 948 in the placebo group). Most of the studies (7 of 13, 53.8%) were published in the United States, and the follow-up period ranged from 4 to 52 weeks. The mean age ranged from 35.7 to 72.4 years in the pregabalin group. The number of female participants, duration of symptoms, conflict of interests (COI), and funding are presented in Table 1. The therapeutic regimens are shown in Supplementary Table 1.

**TABLE 1:** Baseline characteristics of the 14 included studies.

Study	Region	Follow-up	n PGB/CRL^a^/PLA	Mean age PGB/CRL/PLA	Female PGB/CRL/PLA	Duration GAD (years) PGB/CRL/PLA	Doses Pregabalina/Control	COI	Funding
ADAN study (31)	Spain	26w	486/239/NA	47.0/45.2/NA	325/159/NA	NR	186.2 mg/NR	Yes	Yes
Cvjetkovic-Bosnjak et al., 2015 (32)	Serbia	4w	47/60/NA	37.8/37.4/NA	18/41/NA	NR	224 mg/150 mg	No	NR
Feltner et al., 2003 (23)	United States	4w	136/68/67	37.1/39.2/37.8	69/40/34	NR	50,200 mg/2 mg	NR	Yes
Feltner et al. (2008)	United States, Germany	10w	168/NA/170	38.8/NA/38.7	100/NA/93	11/NA/12	450 mg/NA	Yes	Yes
Hadley et al., 2012 (29)	United States	52w	56/NA/50	40.1/NA/43.5	42/NA/34	5.2/NA/7.7	381 mg/NA	Yes	Yes
Kasper et al., 2009 (33)	Austria, Italy, United States, Canada, Germany	8w	121/125/128	39.5/42.6/40.2	77/73/78	3.2/4.0/4.6	150–600 mg/75–225 mg	Yes	Yes
Kasper et al., 2014 (27)	Austria, Spain, United States	26w	412/203/NA	41.5/42.6/NA	252/12/NA	2.2/2.4/NA	150–300,450–600 mg/3–4 mg	Yes	Yes
Montgomery et al., 2006 (35)	United Kingdom	6w	207/113/101	43.5/46.0/43.0	129/73/59	1.6/1.4/1.7	400,600 mg/75 mg/	Yes	Yes
Montgomery et al., 2008 (13)	United Kingdom	8w	177/NA/96	72.4/NA/72.2	140/NA/72	NR	270 mg/NA	Yes	Yes
Pande et al., 2003 (28)	United States	4w	139/68/69	36.7/33.9/35.7	74/43/47	NR	150,600 mg/6 mg	NR	NR
Rickels et al., 2005 (25)	United States	4w	270/93/91	38.3/40.0/41.0	171/61/57	12.7/12.0/13.0	300,450 and 600 mg/1.5 mg	Yes	Yes
Rickels et al., 2012 (30)	United States	8w	180/NA/176	43.7/NA/43.5	129/NA/115	NR	300–600 mg/NA	Yes	Yes
Silva Miguel et al., 2013 (14)	Portugal	8w	121/125/NA	NR	NR	NR	300–600 mg/75–225 mg	Yes	Yes
Vera-Llonch et al., 2010 (34)	Spain/United States	52w	130/130/NA	NR	NR	NR	300–600 mg/75–225 mg	NR	Yes

^a^ The control group comprised patients treated with benzodiazepines or SSRI/SNRI; COI, conflict of interests; CRL, control; NA, not applicable; NR, not reported; PGB, pregabalin; PLA, placebo.

### 3.4 HAM-A

At 2 weeks, the HAM-A global score showed greater efficacy in the pregabalin group (MD −1.23, 95% CI −1.79 to −0.66; participants = 4,232; studies = 23; I2 = 99%). The higher dose of pregabalin (>300 mg) showed significant differences (MD −1.64, 95% CI −2.32 to −0.96; participants = 3,035; studies = 15; I2 = 100%), but no significant differences were found with doses below 300 mg (MD −0.33, 95% CI −2.18 to 1.51; participants = 1,197; studies = 8; I2 = 96%). Table 2 presents the results comparing pregabalin with placebo, benzodiazepines, and SSRIs/SNRIs. Both high and low doses of pregabalin showed significant improvement compared to placebo. Benzodiazepines demonstrated greater reduction in the HAM-A scale compared to low doses of pregabalin. However, no significant differences were found between benzodiazepines and high doses of pregabalin. However, both doses of pregabalin showed significant superiority compared to SSRIs/SNRIs.

**TABLE 2:** Hamilton Anxiety Rating Scale (HAM-A) at different follow-up periods.

Effect size	n studies	n participants	Random effect model (OR 95% CI)	I^2^ (%)	*P*-value
HAM-A 2 weeks					
Pregabalin >300 mg vs. Placebo	8	1,669	MD −2.78, 95% CI −3.46 to −2.11	99	<0.00001
Pregabalin <300 mg vs. Placebo	4	681	MD −1.73, 95% CI −2.34 to −1.12	0	<0.00001
Pregabalin >300 mg vs. Benzodiazepines	4	687	MD −0.25, 95% CI −0.70 to 0.20	97	0.28
Pregabalin <300 mg vs. Benzodiazepines	3	409	MD 2.36, 95% CI 0.76 to 3.96	81	0.004
Pregabalin >300 mg vs. SSRI/SNRI	3	679	MD −1.04, 95% CI −1.31 to −0.77	8	<0.00001
Pregabalin <300 mg vs. SSRI/SNRI	1	107	MD −2.80, 95% CI −3.24 to −2.36	0	0.005
HAM-A 4 weeks					
Pregabalin >300 mg vs. Placebo	8	1,605	MD −2.61, 95% CI −3.20 to −2.03	98	<0.00001
Pregabalin <300 mg vs. Placebo	4	681	MD −2.47, 95% CI −3.43 to −1.51	58	<0.00001
Pregabalin >300 mg vs. Benzodiazepines	4	687	MD −0.40, 95% CI −1.06 to 0.26	99	0.23
Pregabalin <300 mg vs. Benzodiazepines	3	409	MD 2.04, 95% CI −0.33 to 4.41	91	0.09
Pregabalin >300 mg vs. SSRI/SNRI	3	679	MD 0.11, 95% CI −1.49 to 1.71	90	0.89
Pregabalin <300 mg vs. SSRI/SNRI	1	107	MD 0.30, 95% CI 0.09 to 0.51	0	0.004
HAM-A 8 weeks					
Pregabalin <300 mg vs. Placebo	3	840	MD −2.82, 95% CI −4.98 to −0.66	100	0.01
Pregabalin >300 mg vs. Placebo	1	273	MD −3.20, 95% CI −4.28 to −2.12	0	<0.00001
Pregabalin >300 mg vs. SSRI/SNRI	1	246	MD −0.90, 95% CI −1.00 to −0.80	0	<0.00001
HAM-A 12 weeks					
Pregabalin >300 mg vs. Benzodiazepines	1	385	MD 0.10, 95% CI −1.42 to 1.62	0	0.9
Pregabalin <300 mg vs. Benzodiazepines	2	371	MD 1.10, 95% CI −0.45 to 2.65	0	0.16
Pregabalin <300 mg vs. SSRI/SNRI	1	1,546	MD 1.20, 95% CI 0.41 to 1.99	0	0.003
HAM-A 52 weeks					
Pregabalin >300 mg vs. Placebo	1	106	MD −5.00, 95% CI −7.55 to −2.45	0	0.0001
Pregabalin <300 mg vs. SSRI/SNRI	1	725	MD −3.00, 95% CI −4.09 to −1.91	100	<0.00001

At 4 weeks, the HAM-A global score showed a greater reduction in the pregabalin group (MD −1.12, 95% CI −1.60 to −0.63; participants = 4,168; studies = 23; I2 = 99%), with differences in favor of pregabalin at doses over 300 mg (MD −1.46, 95% CI −2.01 to −0.91; participants = 2,971; studies = 15; I2 = 99%), and no differences were found between groups with doses below 300 mg (MD −0.45, 95% CI −1.91 to 1.01; participants = 1,197; studies = 8; I2 = 95%). Table 2 presents the results comparing pregabalin with placebo, benzodiazepines, and SSRIs/SNRIs separately. Both doses of pregabalin showed significant superiority compared to placebo. No significant differences were found compared with benzodiazepines at either dose. SSRIs/SNRIs were superior to low doses of pregabalin, but no differences were found with pregabalin at high doses.

At 8 weeks, the HAM-A global score showed greater improvement in the pregabalin group (MD −2.50, 95% CI −4.21 to −0.79; participants = 1,359; studies = 5; I2 = 100%). Doses below 300 mg and above 300 mg showed greater benefit in the pregabalin group (MD −2.33, 95% CI −4.24 to −0.42; participants = 1,086; studies = 4; I2 = 100%) and (MD 1.18, 95% CI 0.48 to 1.88; participants = 1,917; studies = 2; I2 = 0%), respectively. Pregabalin was superior to placebo and SSRIs/SNRIs when divided by control type (Table 2).

At 12 weeks, the HAM-A global score showed lower scores in the pregabalin group (MD 0.99, 95% CI 0.35 to 1.63; participants = 2,302; studies = 3; I2 = 0%), with no differences observed with the higher dose of 300 mg (MD 0.10, 95% CI −1.42 to 1.62; participants = 385; studies = 1; I2 = 0%), and pregabalin showing higher scores on the HAM-A global scale compared to the control group with doses below 300 mg (MD 1.18, 95% CI 0.48 to 1.88; participants = 1,917; studies = 2; I2 = 0%). At 12 weeks, no differences were found between pregabalin and benzodiazepines for either dose. SSRIs/SNRIs were superior to low doses of pregabalin (Table 2).

At 6 months to 1 year, the HAM-A score showed significantly more favorable results in the pregabalin group (MD −3.31, 95% CI −4.30 to −2.31; participants = 831; studies = 2; I2 = 50%). Both doses above 300 mg and doses below 300 mg showed significantly greater improvement in the pregabalin group: (MD −5.00, 95% CI −7.55 to −2.45; participants = 106; studies = 1; I2 = 0%) and (MD −3.00, 95% CI −4.09 to −1.91; participants = 725; studies = 1; I2 = 100%), respectively. Pregabalin showed superiority over placebo at high doses and superiority over SSRIs/SNRIs at low doses (Table 2).

**FIGURE 3:**
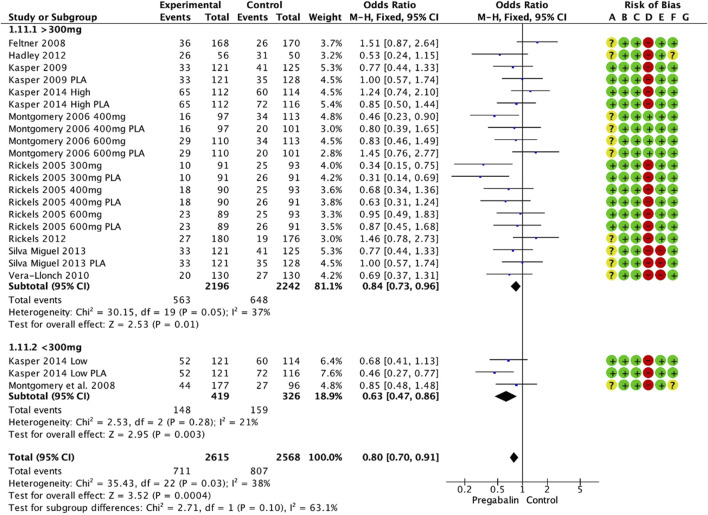
Forest plot depicting response rate based on HAM-A scale. The response rate was significantly higher in the pregabalin group (OR: 1.51; 95% CI 1.31: 1.75).

Independently from study duration, the response rate on the HAM-A scale was significantly higher in the pregabalin group (OR 1.51, 95% CI 1.31 to 1.75; participants = 3,092; studies = 13; I2 = 6%) (Figure 3). There were significant differences in favor of pregabalin both with doses above 300 mg and doses below 300 mg (OR 1.79, 95% CI 1.45 to 2.22; participants = 1,397; studies = 7; I2 = 0%) and (OR 1.32, 95% CI 1.08 to 1.60; participants = 1,695; studies = 6; I2 = 0%). When compared to different control groups, pregabalin showed significant superiority over placebo at high doses (OR 1.99, 95% CI 1.51 to 2.61; participants = 872; studies = 4; I2 = 0%) and low doses (OR 1.60, 95% CI 1.13 to 2.28; participants = 535; studies = 3; I2 = 0%). It also showed superiority over benzodiazepines at high doses (OR 1.52, 95% CI 1.08 to 2.15; participants = 525; studies = 3; I2 = 0%). However, only the low dose of pregabalin could be compared with SSRIs/SNRIs, and no significant differences were found (OR 1.26, 95% CI 0.97 to 1.65; participants = 902; studies = 1; I2 = 0%).

### 3.5 HAM-A psychic

When the HAM-A scale was divided according to the psychic subdomain, the pregabalin group showed higher overall satisfaction scores at the end of the follow-up (MD −0.92, 95% CI −1.49 to −0.35; participants = 3,361; studies = 18; I2 = 100%). Doses above 300 mg showed significant differences in favor of pregabalin (MD −1.10, 95% CI −1.75 to −0.46; participants = 2,706; studies = 14; I2 = 100%), while doses below 300 mg did not show differences between the groups (MD −0.19, 95% CI −1.36 to 0.98; participants = 655; studies = 4; I2 = 86%). Both doses of pregabalin showed significant superiority over placebo: (MD −2.05, 95% CI −2.56 to −1.53; participants = 1,340; studies = 7; I2 = 99%) and (MD −1.21, 95% CI −1.82 to −0.59; participants = 411; studies = 2; I2 = 12%), respectively. In contrast, high doses of pregabalin showed significant superiority compared to benzodiazepines (MD −0.36, 95% CI −0.59, −0.14; participants, 687; studies, 4; I2 = 87%). There were no significant differences compared with SSRIs/SNRIs (MD 0.17, 95% CI −0.68 to 1.03; participants = 786; studies = 4; I2 = 99%).

### 3.6 HAM-A somatic

In contrast, the HAM-A somatic domain showed better values in the pregabalin group (MD −0.64, 95% CI −1.04 to −0.25; participants, 3,361; studies, 18; I2 = 99%). The group receiving doses above 300 mg showed significant differences in favor of pregabalin (MD −0.79, 95% CI −1.23 to −0.36; participants = 2,706; studies = 14; I2 = 99%), and the group receiving doses below 300 mg did not show significant differences (MD 0.03, 95% CI −0.91 to 0.97; participants = 655; studies = 4; I2 = 77%). Both doses of pregabalin showed significant superiority over placebo: (MD -1.31, 95% CI -1.86 to −0.76; participants = 1,340; studies = 7; I2 = 99%) and (MD −0.80, 95% CI −1.43 to −0.17; participants = 411; studies = 2; I2 = 0%), respectively. In contrast, low doses of pregabalin showed significant superiority compared to benzodiazepines (MD 1.00, 95% CI 0.01 to 1.99; participants = 137; studies = 1; I2 = 0%). There were no significant differences compared with SSRIs/SNRIs (MD −0.14, 95% CI −1.03 to 0.76; participants = 786; studies = 4; I2 = 99%, p = 0.77).

### 3.7 CGI-I

**FIGURE 4:**
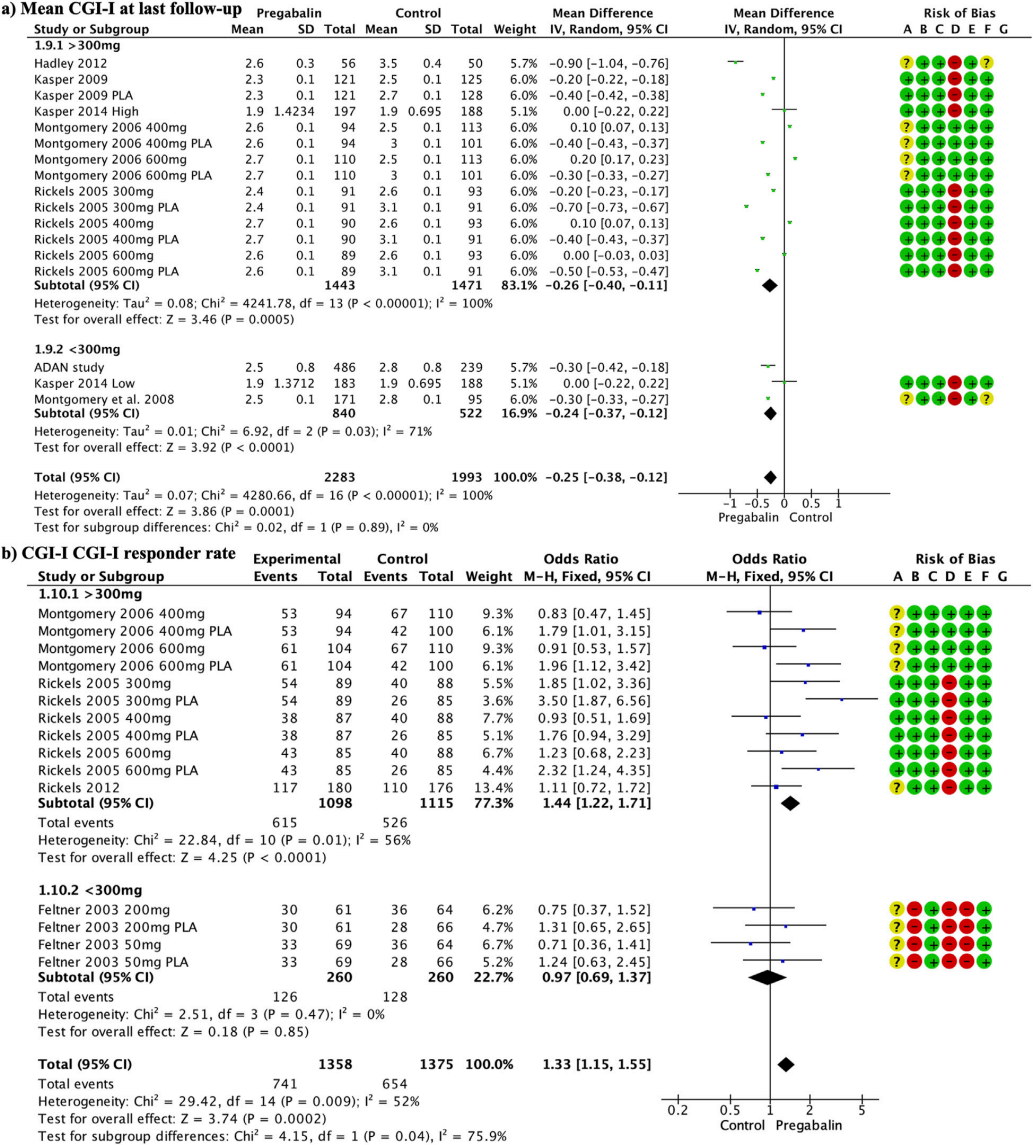
Forest plot illustrating a significant improvement in CGI-I at the end of follow-up in the pregabalin group **(A)**. The response rate to CGI-I at the end of the follow-up was significantly higher in the pregabalin group (OR 1.33, 95% CI 1.15–1.55) **(B)**.

CGI-I at the end of the follow-up showed significant improvement in the pregabalin group overall (MD −0.25, 95% CI −0.38 to −0.12; participants = 4,276; studies = 17; I2 = 100%) (Figure 4A). Doses above 300 mg and below 300 mg showed differences in favor of pregabalin: (MD −0.26, 95% CI −0.40 to −0.11; participants = 2,914; studies = 14; I2 = 100%) and (MD −0.24, 95% CI −0.37 to −0.12; participants = 1,362; studies = 3; I2 = 71%). Both doses of pregabalin showed significant superiority over placebo: (MD −0.50, 95% CI −0.61 to −0.40; participants = 1,304; studies = 7; I2 = 99%) and (MD −0.30, 95% CI −0.33 to −0.27; participants = 266; studies = 1; I2 = 0%). There were no significant differences compared with benzodiazepines (MD −0.07, 95% CI −0.20, 0.06; participants, 2,030; studies, 6; I2 = 98%). Low doses of pregabalin were superior to SSRIs/SNRIs (MD −0.30, 95% CI −0.42 to −0.18; participants = 725; studies = 1; I2 = 0%).

The response rate to CGI-I at the end of the follow-up was significantly higher in the pregabalin group (OR 1.33, 95% CI 1.15 to 1.55; participants = 2,733; studies = 15; I2 = 52%) (Figure 4B). Doses above 300 mg showed significant differences in favor of pregabalin (OR 1.44, 95% CI 1.22 to 1.71; participants = 2,213; studies = 11; I2 = 56%), whereas lower doses did not show significant differences (OR 0.97, 95% CI 0.69 to 1.37; participants = 520; studies = 4; I2 = 0%). High doses of pregabalin showed significant superiority over placebo (OR 1.80, 95% CI 1.43 to 2.25; participants = 1,270; studies = 6; I2 = 49%), whereas no significant differences were found compared with benzodiazepines or SSRIs/SNRIs (OR 1.07, 95% CI 0.81 to 1.41; participants = 783; studies = 5; I2 = 33%) and (OR 0.87, 95% CI 0.59 to 1.29; participants = 418; I2 = 0%), respectively.

### 3.8 Discontinuation

**FIGURE 5:**
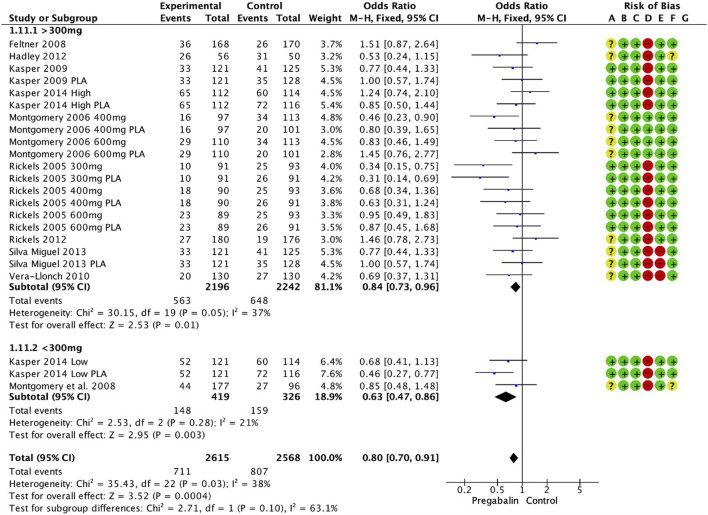
A forest plot demonstrating a significantly lower discontinuation rate in the pregabalin group (OR 0.80, 95% CI 0.70, 0.91).

The discontinuation rate was significantly lower in the pregabalin group (OR 0.80, 95% CI 0.70 to 0.91; participants = 5,183; studies = 23; I2 = 38%) (Figure 5). There were significant differences in favor of pregabalin for doses above 300 mg and below 300 mg: (OR 0.84, 95% CI 0.73 to 0.96; participants = 4,438; studies = 20; I2 = 37%) and (OR 0.63, 95% CI 0.47 to 0.86; participants = 745; studies = 3; I2 = 21%), respectively. The low dose of pregabalin showed a significantly lower discontinuation rate than placebo (OR 0.61, 95% CI 0.42 to 0.89; participants = 510; studies = 2; I2 = 59%). Additionally, the low dose of pregabalin showed a lower discontinuation rate compared to SSRIs/SNRIs (OR 0.68, 95% CI 0.41 to 1.13; participants = 235; studies = 1; I2 = 0%). There were no differences compared with benzodiazepines (OR 0.77, 95% CI 0.59 to 1.01; participants = 1,010; studies = 5; I2 = 50%).

Discontinuation due to adverse events did not differ between the groups (OR 0.90, 95% CI 0.76 to 1.07; participants = 5,183; studies = 23; I2 = 46%). Doses above 300 mg had a significantly lower discontinuation rate (OR 0.82, 95% CI 0.68 to 0.99; participants = 4,438; studies = 20; I2 = 46%), while there were no differences in doses below 300 mg (OR 1.43, 95% CI 0.92 to 2.21; participants = 745; studies = 3; I2 = 0%). The discontinuation rate due to adverse events with high doses of pregabalin was significantly lower for pregabalin than for SSRIs/SNRIs (OR 0.57, 95% CI 0.41 to 0.79; participants = 1,185; studies = 5; I2 = 0). There were no differences when compared with placebo (OR 1.11, 95% CI 0.86 to 1.45; participants = 2,739; studies = 12; I2 = 28%) or benzodiazepines (OR 0.91, 95% CI 0.62 to 1.34; participants = 1,010; studies = 5; I2 = 51%).

The discontinuation rate due to a lack of efficacy was significantly lower in the pregabalin group (OR 0.58, 95% CI 0.44 to 0.77; participants, 4,845; studies, 22; I2 = 0%). High doses showed a significantly lower discontinuation rate in the pregabalin group (OR 0.54, 95% CI 0.39 to 0.75; participants = 4,100; studies = 19; I2 = 0%), whereas low doses did not show significant differences (OR 0.68, 95% CI 0.41 to 1.13; participants = 745; studies = 3; I2 = 0%). The discontinuation rate due to lack of efficacy was significantly lower with pregabalin than with placebo (OR 0.44, 95% CI 0.31 to 0.63; participants = 2,650; studies = 12; I2 = 0%). There were no differences between pregabalin and benzodiazepines (OR 0.76, 95% CI 0.43 to 1.35; participants = 1,010; studies = 5; I2 = 0%) or SSRIs/SNRIs (OR 1.19, 95% CI 0.57 to 2.46; participants = 1,185; studies = 5; I2 = 0%).

### 3.9 Adverse events

Adverse events are shown in Tables 3, 4. Compared to SSRI/SNRI, high-dose pregabalin (Table 3) showed a significantly lower rate of nausea (OR 0.36, 95% CI 0.24–0.54), blurred vision (OR 0.32, 95% CI 0.15–0.71), asthenia (OR 0.32, 95% CI 0.15–0.71), insomnia (OR 0.34, 95% CI 0.15–0.74), and ataxia (OR 0.36, 95% CI 0.14–0.94). In contrast, SSRI/SNRI showed a significantly lower rate of somnolence (OR 4.17, 95% CI 1.20–14.55) and dizziness (OR 2.35, 95% CI 1.55–3.57). Low doses of pregabalin could not be compared with those of SSRIs/SNRIs.

**TABLE 3:** Adverse events of pregabalin at doses higher than 300 mg.

Effect size	n studies	n participants	Random effect model (OR 95% CI)	I^2^ (%)	*P*-value
SSRI/SNRI					
Somnolence	3	679	OR 4.17, 95% CI 1.20 to 14.55	56	0.02
Dizziness	3	679	OR 2.35, 95% CI 1.55 to 3.57	0	<0.0001
Dry mouth	3	679	OR 0.77, 95% CI 0.44 to 1.36	0	0.37
Infection	2	433	OR 4.32, 95% CI 1.71 to 10.90	0	0.02
Nausea	3	679	OR 0.36, 95% CI 0.24 to 0.54	0	<0.0001
Blurred vision	2	433	OR 0.32, 95% CI 0.15 to 0.71	0	0.005
Asthenia	2	433	OR 0.32, 95% CI 0.15 to 0.71	0	0.005
Consitpation	3	679	OR 0.97, 95% CI 0.51 to 1.85	0	0.93
Headache	3	679	OR 0.98, 95% CI 0.60 to 1.58	0	0.93
Diarrhea	2	433	OR 0.98, 95% CI 0.39 to 2.47	0	0.97
Insomnia	3	679	OR 0.34, 95% CI 0.15 to 0.74	0	0.006
Ataxia	2	433	OR 0.36, 95% CI 0.14 to 0.94	0	0.04
Benzodiazepines					
Somnolence	5	1,096	OR 0.69, 95% CI 0.53 to 0.89	0	0.0005
Dizziness	5	1,096	OR 2.90, 95% CI 2.16 to 3.88	0	<0.00001
Dry mouth	5	1,096	OR 3.67, 95% CI 1.85 to 7.30	51	0.0002
Incoordination	4	687	OR 2.01, 95% CI 0.60 to 6.73	73	0.26
Infection	5	1,096	OR 1.87, 95% CI 1.19 to 2.94	0	0.007
Nausea	5	1,096	OR 1.11, 95% CI 0.76 to 1.62	0	0.6
Blurred vision	4	687	OR 0.58, 95% CI 0.36 to 0.93	0	0.02
Asthenia	4	687	OR 0.58, 95% CI 0.36 to 0.93	0	0.02
Consitpation	5	1,096	OR 2.15, 95% CI 1.07 to 4.32	11	0.03
Headache	2	547	OR 1.64, 95% CI 0.75 to 3.56	54	0.22
Diarrhea	2	547	OR 1.93, 95% CI 0.64 to 5.83	41	0.24
Insomnia	2	547	OR 0.79, 95% CI 0.16 to 3.81	56	0.77
Pain	2	547	OR 0.94, 95% CI 0.53 to 1.65	0	0.82

**TABLE 4:** Adverse events of pregabalin at doses lower than 300 mg.

Effect size	n studies	n participants	Random effect model (OR 95% CI)	I^2^ (%)	*P*-value
Benzodiazepines					
Somnolence	4	818	OR 0.39, 95% CI 0.18 to 0.83	81	0.01
Dizziness	4	818	OR 1.31, 95% CI 0.78 to 2.22	46	0.31
Dry mouth	4	818	OR 1.41, 95% CI 0.65 to 3.07	42	0.39
Infection	4	818	OR 1.71, 95% CI 0.74 to 3.93	6	0.21
Nausea	4	818	OR 0.65, 95% CI 0.41 to 1.02	0	0.06
Asthenia	3	409	OR 0.39, 95% CI 0.18 to 0.86	0	0.02
Consitpation	2	546	OR 1.01, 95% CI 0.18 to 5.76	45	0.99
Headache	4	818	OR 1.54, 95% CI 1.06 to 2.23	0	0.02
Amblyopia	3	409	OR 2.78, 95% CI 0.63 to 12.31	11	0.18
Accidental injury	2	272	OR 3.02, 95% CI 0.59 to 15.52	0	0.19
Rhinitis	2	272	OR 9.72, 95% CI 0.51 to 184.22	0	0.13
Amnesia	3	409	OR 0.44, 95% CI 0.15 to 1.30	0	0.14
Diarrhea	4	818	OR 1.50, 95% CI 0.72 to 3.13	0	0.27
Confusion	2	272	OR 0.15, 95% CI 0.03 to 0.70	0	0.02
Insomnia	2	546	OR 0.65, 95% CI 0.39 to 1.09	0	0.11
Vomiting	4	818	OR 1.37, 95% CI 0.69 to 2.72	51	0.76

On the other hand, high doses of pregabalin (Table 4) showed a significantly lower rate of somnolence (OR 0.69, 95% CI 0.53–0.89), blurred vision (OR 0.58, 95% CI 0.36–0.93), and asthenia (OR 0.58, 95% CI 0.36–0.93) compared to benzodiazepines. However, benzodiazepines showed a significantly lower rate of dizziness (OR 2.90, 95% CI, 2.16–3.88), dry mouth (OR 3.67, 95% CI, 1.85–7.30), infection (OR 1.87, 95% CI 1.19–2.94), and constipation (OR 2.15, 95% CI, 1.07–4.32). Low doses of pregabalin (Table 4) resulted in a significantly lower rate of somnolence (OR 0.39, 95% CI 0.18–0.83), asthenia (OR 0.39, 95% CI 0.18–0.86), and confusion (OR 0.15, 95% CI 0.03–0.70) compared to benzodiazepines. In contrast, benzodiazepines showed a lower incidence of headache than low-dose pregabalin (OR 1.54, 95% CI 1.06–2.23).

### 3.10 Costs

**FIGURE 6:**
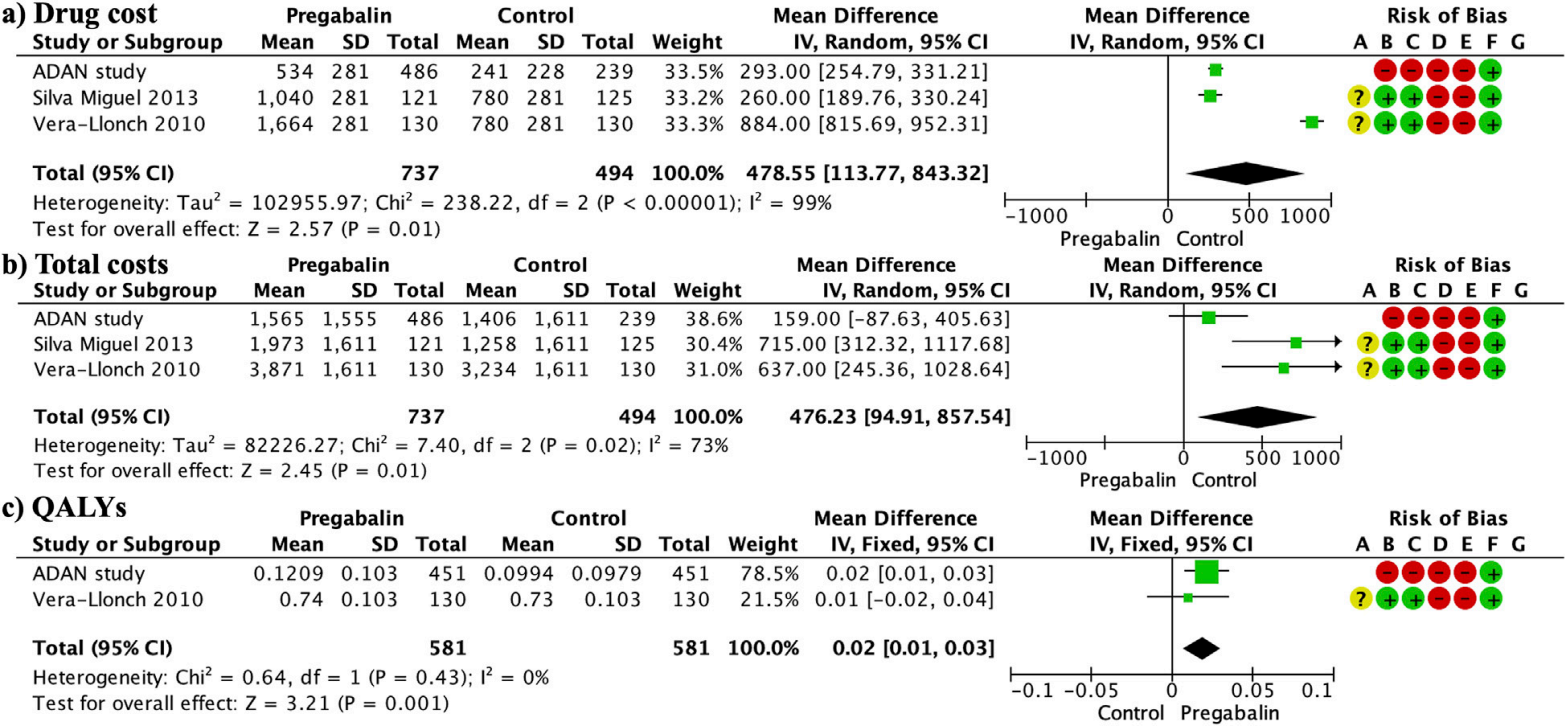
Forest plots presenting the cost analysis. The total costs were significantly higher in the pregabalin group than in the SSRI/SNRI group **(A)**. Furthermore, drug costs were significantly higher in the pregabalin group than in the SSRI/SNRI group **(B)**. QALYs were significantly higher in the pregabalin group than in the SSRI/SNRI group **(C)**.

The total cost was significantly higher in the pregabalin group than in the SSRI/SNRI group (MD 476.23, 95% CI 94.91 to 857.54; participants = 1,231; studies = 3; I2 = 73%) (Figure 6A). This was mainly due to the significantly higher drug costs in the pregabalin group than in the SSRI/SNRI group (MD 478.55, 95% CI 113.77 843.32; participants = 1,231; studies = 3; I2 = 99%) (Figure 6B). However, the cost-effectiveness, evaluated using QALYs, was significantly higher in the pregabalin group than in the SSRI/SNRI group (MD 0.02, 95% CI 0.01 to 0.03; participants = 1,162; studies = 2; I2 = 0%) (Figure 6C).

### 3.11 Publication bias

**FIGURE 7:**
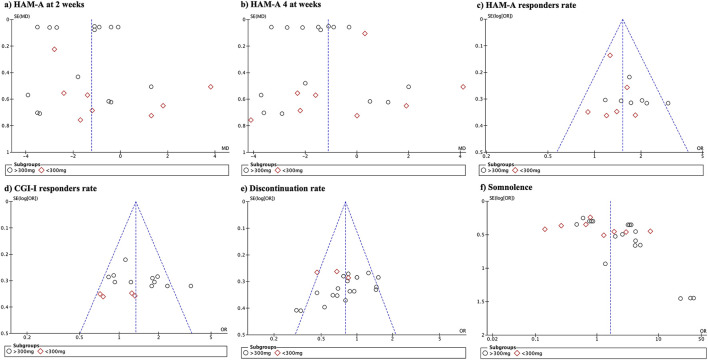
Funnel plot demonstrating the presence of publication bias.

Publication bias (Figure 7) was high for the HAM-A mean difference at different follow-up periods and adverse events. No publication bias was observed in the remaining variables.

### 3.12 GRADE

Table 5 presents the results of the GRADE assessment. There was a high level of certainty for the responder rate on the HAM-A and CGI-I scales as well as for the discontinuation rate. Regarding the mean difference in HAM-A scores at 8 weeks, the certainty was moderate due to increased publication bias. The short-term HAM-A variable (2 weeks) showed low certainty owing to high publication bias and wide variability in the results.

**TABLE 5:** GRADE assessment of the quality of the evidence and the strength of the recommendations.

Certainty assessment							No of patients		Effect		Certainty	Importance
No of studies	Study design	Risk of bias	Inconsistency	Indirectness	Imprecision	Other considerations	Clinical	Placebo	Relative (95% CI)	Absolute (95% CI)		
HAM-A 2 weeks												
23	Randomised trials	Not serious	Serious^a^	Not serious	Not serious	Publication bias strongly suspected^b^	2,136	2,096	—	MD 1.23 lower (1.79 lower to 0.66 lower)	⊕⊕⊕○○○ Low	CRITICAL
HAM-A 8 weeks												
5	Randomised trials	Not serious	Not serious	Not serious	Serious^c^	Publication bias strongly suspected strong association^b^	713	646	—	MD 2.5 lower (4.21 lower to 0.79 lower)	⊕⊕⊕○ Moderate	CRITICAL
HAM-A 52 weeks/6 months												
2	Randomised trials	Serious^d^	Not serious	Not serious	Not serious	Strong association	542	289	—	MD 3.31 lower (4.3 lower to 2.31 lower)	⊕⊕⊕⊕ High	CRITICAL
HAM-A responder rate LFU												
13	Randomised trials	Serious^d^	Not serious	Not serious	Not serious	Strong association	887/1,590 (55.8%)	689/1,502 (45.9%)	OR 1.51 (1.31–1.75)	103 more per 1,000 (from 67 more to 139 more)	⊕⊕⊕⊕ High	CRITICAL
CGI-I responder rate LFU												
15	Randomised trials	Not serious	Not serious	Not serious	Not serious	Strong association	741/1,358 (54.6%)	654/1,375 (47.6%)	OR 1.33 (1.15–1.55)	71 more per 1,000 (from 35 more to 109 more)	⊕⊕⊕⊕ High	CRITICAL
n Discontinued												
23	Randomised trials	Not serious	Not serious	Not serious	Not serious	None	711/2,615 (27.2%)	807/2,568 (31.4%)	OR 0.80 (0.70–0.91)	46 fewer per 1,000 (from 71 fewer to 20 fewer)	⊕⊕⊕⊕ High	CRITICAL
n Disc: Adverse events												
23	Randomised trials	Not serious	Not serious	Not serious	Not serious	None	275/2,615 (10.5%)	297/2,568 (11.6%)	OR 0.90 (0.76–1.07)	10 fewer per 1,000 (from 25 fewer to 7 more)	⊕⊕⊕⊕ High	CRITICAL

a, The results showed a large variability; b, Suspected publication bias through funnel plots; c, Wide confidence intervals; CI, confidence interval; d, High proportion of included studies with high risk of bias; MD, mean difference; OR, odds ratio.

## 4 Discussion

This meta-analysis found that most measures of anxiety improvement favored pregabalin treatment, although the differences were modest in some cases. Scores on the HAM-A and CGI scales consistently showed more favorable outcomes with pregabalin than with placebo, with doses higher than 300 mg driving a significant portion of this effect. The response rates on the HAM-A scale were generally higher for pregabalin and discontinuation rates were significantly lower. While discontinuation due to adverse events did not differ clearly, the rates of discontinuation due to lack of efficacy seemed lower with pregabalin than with SSRIs/SNRIs. Measures of sleep disturbance also showed better outcomes in the pregabalin group. The safety profiles differed between the drugs. Higher doses of pregabalin resulted in lower rates of nausea, blurred vision, asthenia, and insomnia than SSRI/SNRI, but higher rates of somnolence. Compared with benzodiazepines, pregabalin had less somnolence, visual effects, and asthenia, although at low doses, there was a higher incidence of headache. Benzodiazepines were associated with a decreased risk of dizziness, dry mouth, infections, and constipation. Although the costs were higher for pregabalin, QALYs demonstrated superior efficacy in the pregabalin group. It is important to consider that most comparisons between pregabalin and other treatments are based on short- or medium-term studies. Notably, in longer follow-up periods, such as 6 months or 1 year, the differences were even more pronounced, although only two studies were included. Overall, significant differences in favor of pregabalin were observed, particularly at doses >300 mg. When evaluating the Hamilton Anxiety Rating Scale (HAM-A), not only was the mean difference considered, as it could be influenced by extreme values but also the percentage of patients who achieved clinically relevant outcomes or the response rate. Pregabalin demonstrated greater efficacy in response rates for doses below and above 300 mg, with 56% of pregabalin-treated patients experiencing clinically relevant improvement compared to 46% in the control group. Similarly, the Clinical Global Impressions-Improvement (CGI-I) scale showed that both high and low doses of pregabalin had a mean difference favoring pregabalin. However, significant differences in the response rates were observed only at high doses. Overall, the response rate in the pregabalin group according to CGI-I was 55% compared with 48% in the control group. When analyzing the HAM-A subdomains (psychic and somatic), a significantly greater efficacy of both pregabalin doses was observed, without differences between the lower doses. It is important to highlight that the evaluation of these subdomains and the improvement in the CGI-I were carried out at the end of the follow-up, mixing follow-up periods generally between 4 and 8 weeks, without being able to further subdivision of the follow-up times. Although visual inspection of the forest plots in relation to follow-up periods suggested an increase in differences favoring pregabalin over time, this was a visual observation of trends. These findings were also confirmed by the discontinuation rate due to lack of efficacy, which was 3.5% in the pregabalin group and 5.7% in the comparison group. Although the Hamilton Anxiety Rating Scale (HAM-A) has multiple subdomains, only psychic and somatic aspects were analyzed in the meta-analyses. The efficacy of pregabalin on the global HAM-A score may also be attributed to its effect on other associated symptoms such as depressive mood, sleep disturbances, and somatic symptoms. Notably, pregabalin’s impact on the somatic components of anxiety is particularly significant, as evidenced by its efficacy in treating neuropathic pain, postoperative pain, and in reducing nausea and vomiting (Montgomery et al., 2006; Onakpoya et al., 2019; Hung et al., 2022) In comparing adverse events between anxiety disorder treatments, pregabalin has been shown to have a lower incidence compared to SSRIs, SNRIs, and benzodiazepines, despite causing greater somnolence, dizziness, dry mouth, and headaches, especially at higher doses. Notably, these reactions did not significantly influence the decision to discontinue pregabalin, with discontinuation rates of 27% for pregabalin and 31% for the control group. Pande et al. suggested that accelerated dose titration in clinical trials might be the source of initial somnolence and dizziness, indicating that personalized and slower dose titration could mitigate these effects (Pande et al., 2003). In addition, pregabalin has shown promising results in the treatment of insomnia, which affects 60%–70% of patients with generalized anxiety disorder (GAD) and significantly affects their quality of life (Greist et al., 2011). Pregabalin exhibited an incidence of insomnia that was three times lower than that of SSRIs and SNRIs. In this study, both low and high doses of pregabalin showed significant improvements in sleep scales compared to SSRIs, SNRIs, and placebo. Pregabalin improves sleep through various physiological mechanisms (Feltner et al., 2011). First, it increases the proportion of slow-wave sleep (SWS), which plays a crucial role in body restoration and memory consolidation. This improvement in SWS may have positive implications for physical and cognitive wellbeing. Second, pregabalin did not significantly affect stage 2 sleep and maintained a more natural sleep pattern. Additionally, it reduces the frequency of short and long awakenings, leading to an uninterrupted sleep experience. It also improves total sleep time, reduces the time to fall asleep, and increases sleep efficiency, contributing to a more restorative night. Subjectively, patients report finding it easier to fall asleep and experiencing improved sleep quality. On the other hand, when analyzing costs, pregabalin was found to be significantly more expensive than SSRI/SNRIs, resulting in a higher total cost. The higher cost of pregabalin compared to SSRIs/SNRIs may be attributed to the fact that, during the clinical trials, the comparator groups (benzodiazepines and SSRIs/SNRIs) were already available as generic medications. Notably, the recent loss of patent protection for pregabalin is expected to lead to a reduction in these costs as generic versions become available. However, when comparing the QALYs, pregabalin yielded higher values. Thus, pregabalin is expected to provide more QALYs. While implying a higher initial expenditure, the improvement in QALYs could translate into long-term benefits, such as reductions in costs associated with health complications, hospitalization, or additional treatments. Additionally, improved quality of life could have a positive impact on non-monetary aspects, such as functionality, ability to carry out daily activities, and patients’ emotional wellbeing. In the treatment of generalized anxiety disorder (GAD), the choice between pregabalin and SSRIs/SNRIs should be based on the tolerability profile and target symptoms to be treated. Additionally, pregabalin may be a suitable option for patients requiring quicker effect improvement in anxiety symptoms, as it has demonstrated greater anxiety reduction than SSRIs/SNRIs. Other factors such as sexual dysfunction associated with antidepressants may also be relevant in the choice of treatment. Notably, only one study assessed sexual dysfunction using the Changes in Sexual Function Questionnaire (CSFQ) and analyzed the results separately for males and females. In males, there was no significant difference between pregabalin and venlafaxine, whereas in females, pregabalin showed modest improvement compared to venlafaxine (Kasper et al., 2009). Furthermore, the tolerability of pregabalin at low doses, reflected in a lower discontinuation rate compared to SSRIs/SNRIs, may make it appropriate for patients sensitive to side effects or long-term adherence issues. Pregabalin at high doses has shown a higher incidence of dizziness, dry mouth, infections, and constipation than benzodiazepines but offers the advantage of a lower incidence of somnolence, blurred vision, and asthenia, which may be preferable for patients needing to maintain a high level of alertness during the day. Low doses of pregabalin, on the other hand, are associated with less somnolence, asthenia and confusion than benzodiazepines, but with an increase in headache frequency. Although pregabalin does not differ significantly from benzodiazepines in the overall decrease in anxiety according to the HAMA-A scale, high doses are superior in treating the psychic symptoms of anxiety, and low doses show greater efficacy in improving somatic symptoms. This suggests that pregabalin may be particularly beneficial for patients seeking relief focused on psychic and somatic anxiety symptoms and for those who may be more sensitive to common benzodiazepine side effects. Our study aligns with another meta-analysis that compared the efficacy and safety of pregabalin in fibromyalgia. Higher doses of 600 mg were found to have a greater effect on sleep quality, whereas lower doses of 300 mg had fewer adverse events (Schjerning et al., 2018). For specific anxiety disorders, such as generalized social anxiety disorder, pregabalin has been shown to be effective at doses of 450 mg and 600 mg daily (Nutt et al., 2009; Arnold et al., 2015). The effect of pregabalin on depression has primarily been studied in depression associated with fibromyalgia, showing efficacy in pain reduction (Arnold et al., 2015). Karaiskos et al. (2013) reported significant improvement on the Hamilton Depression Rating Scale when pregabalin was added to antidepressant treatment, with a low incidence of adverse events. Olivares et al. (2015) found that in patients with severe depressive symptoms, adding pregabalin to antidepressants or benzodiazepines for 2 weeks significantly improved scores on various scales, positively affecting sleep quality. Additionally, there was a significant decrease in suicidal thoughts and improvement in the ability to concentrate. In patients with schizophrenia and anxiety, pregabalin did not show statistical differences in the total HAM-A14 scale, but there were clinically relevant differences in the psychic subscale of the HAM-A scale and the HAM-A6 scale, which measures the severity of anxiety (Schjerning et al., 2018). Lastly, pregabalin has also been found to have a rapid effect within hours in patients with high anxiety undergoing dental surgery (Nutt et al., 2009).

Finally, this meta-analysis indicates that pregabalin has a low discontinuation syndrome rate, supporting its low risk of potential abuse in real-world clinical settings compared to drugs such as benzodiazepines. Despite concerns regarding pregabalin abuse, it is important to note that most cases involve significantly higher doses and patterns of polysubstance use, which are markedly different from the clinical use of pregabalin in medical practice (Servais et al., 2023; Bonnet and Scherbaum, 2017).

### 4.1 Limitations

Several limitations were encountered during the development of this study that must be considered when interpreting the results. First, missing data had to be handled according to Cochrane guidelines to estimate standard deviations. While studies provided information in graphical form, software (WebPlotDigitizer) was used to extract the necessary data. Additionally, for some variables measured at follow-up, there was a mix of follow-ups conducted between 4 and 8 weeks, requiring adjustments to closer follow-ups. Importantly, the lack of long-term outcomes, for example, beyond 6 months, limits our complete understanding of the long-term effects of pregabalin. Furthermore, some studies did not clearly provide patient numbers in each group, hindering comparative analysis. It was not possible to compare low-dose pregabalin adverse events with SSRI/SNRIs, and some adverse events, such as weight gain, were reported in only one study, preventing their inclusion in the meta-analysis. In addition, studies assessing the effects of different drugs on specific side effects, including weight gain, are insufficient. In conducting subgroup analyses, some subgroups had a limited number of included studies, raising questions about robustness of the results. It would have been interesting to include over 10 studies to enable meta-regressions exploring potential confounders, as in some cases, factors could only be visually studied through graph inspection.

### 4.2 Strengths

This meta-analysis has several significant strengths that contribute to its robustness and relevance. First, it stands out as the most recent study to date, implying that it includes the most up-to-date information on the topic in question. Additionally, notable inclusion of a large number of studies was achieved, increasing the representativeness and generalizability of the results obtained. This wide study inclusion also allowed for greater control of heterogeneity, thus strengthening the internal validity and reliability of the findings. An additional strength of this meta-analysis is the variety of comparisons made. Beyond placebos, first-line drugs, such as benzodiazepines and SSRI/SNRIs, were included, providing a more complete and practical perspective of pregabalin’s efficacy relative to conventional treatments. Another key strength of this study is the presentation of the results. Detailed information on the primary variable of interest across different follow-up periods was provided, allowing for a more accurate evaluation of the temporal evolution of pregabalin effects. In addition, the inclusion criteria were well defined for GAD, and the definitions of response rates used were homogeneous, allowing for more precise comparability between studies. Additionally, an exhaustive exploration of subgroups considering dose and control drug types was performed. This provides greater granularity in the analysis and allows for the identification of possible patterns or differences in treatment responses based on these variables.

## 5 Conclusion

This meta-analysis exhaustively evaluated the efficacy and safety of pregabalin for the treatment of GAD compared with placebo, benzodiazepines, and SSRIs/SNRIs. The results showed that pregabalin significantly reduced HAM-A scores consistently in the short, medium, and long terms, with a clinically relevant magnitude of effect. This improvement was evident in both the psychological and somatic domains of anxiety. The effects were particularly beneficial for doses above 300 mg, with this dose driving many of the differences compared to the comparators. It also significantly improved the response rates on the HAM-A and CGI-I scales in most comparisons. This substantially reduced the total discontinuation rates, especially with respect to SSRIs/SNRI. Regarding safety, high doses of pregabalin showed lower rates of nausea, blurred vision, asthenia, and insomnia than SSRIs/SNRIs but higher somnolence in the latter. Benzodiazepines and pregabalin had less somnolence, visual effects, and asthenia, although low doses were linked to higher headaches. Benzodiazepines are associated with a lower risk of dizziness, dry mouth, infections, and constipation. The results point to pregabalin’s value as a prominent therapeutic option within the range of drugs for generalized anxiety disorders. It is an effective and well-tolerated alternative to initial options.

## Data Availability

The original contributions presented in the study are included in the article/Supplementary Material, further inquiries can be directed to the corresponding author.
