# Supplementary material for: Does pregabalin offer potential as a first-line therapy for generalized anxiety disorder? A meta-analysis of efficacy, safety, and cost-effectiveness
Source: Front Pharmacol. 2025 Feb 7;16:1483770. doi: 10.3389/fphar.2025.1483770 (PMC11842937; doi:10.3389/fphar.2025.1483770)
Supplement: Supplementary file 1 [file Supplementaryfile1.docx]

**Supplementary File 1.** PubMed search strategy.

Search: **(pregabalin OR lyrica) AND anxiety**

("pregabalin"[MeSH Terms] OR "pregabalin"[All Fields] OR "pregabalin s"[All Fields] OR "pregabaline"[All Fields] OR ("pregabalin"[MeSH Terms] OR "pregabalin"[All Fields] OR "lyrica"[All Fields] OR "pregabalin s"[All Fields] OR "pregabaline"[All Fields])) AND ("anxiety"[MeSH Terms] OR "anxiety"[All Fields] OR "anxieties"[All Fields] OR "anxiety s"[All Fields])

**Translations**

**pregabalin:** "pregabalin"[MeSH Terms] OR "pregabalin"[All Fields] OR "pregabalin's"[All Fields] OR "pregabaline"[All Fields]

**lyrica:** "pregabalin"[MeSH Terms] OR "pregabalin"[All Fields] OR "lyrica"[All Fields] OR "pregabalin's"[All Fields] OR "pregabaline"[All Fields]

**anxiety:** "anxiety"[MeSH Terms] OR "anxiety"[All Fields] OR "anxieties"[All Fields] OR "anxiety's"[All Fields]
