# Supplementary material for: Does pregabalin offer potential as a first-line therapy for generalized anxiety disorder? A meta-analysis of efficacy, safety, and cost-effectiveness
Source: Front Pharmacol. 2025 Feb 7;16:1483770. doi: 10.3389/fphar.2025.1483770 (PMC11842937; doi:10.3389/fphar.2025.1483770)
Supplement: Supplementary file 2 [file Table1.docx]

| **Study** | **Treatment schemes** | **Free-drug period** |
| --- | --- | --- |
| **Rickels et al. 2005** | Pregabalin: Initiated at 300 mg/d for all 3 dosages; the dosage was titrated to 450 mg/d on day 4; the dosage was titrated to 600 mg/d on day 7. Alprazolam: Initiated at 0.5 mg/d and increased to 1.0 mg/d on day 4 and to 1.5 mg/d on day 7. Study drug was administered in divided dosesusing a 3 times a day schedule. | Patients who met the study enrollment criteria completed a 1-week drug-free screening period |
| **Feltner et al. 2003** | Study medication was titrated during the first 6 days of double-blind treatment, maintaining a constant number of capsules to preserve the blind, until the targeted dose of pregabalin 50 mg tid, pregabalin 200 mg tid, or lorazepam 2 mg tid was reached. | Patients were required to be free of psychotropic medications for 2 weeks (5 weeks for fluoxetine) prior to enrollment. |
| **Kasper et al. 2014** | Initial pregabalin: 150 mg/day. The initial dose of lorazepam was 2 mg/day. The dose was increased during the first 3 weeks. After the dose escalation, patients received pregabalin at high dose (450-600 mg/day), pregabalin at low dose (150-300 mg/day), or lorazepam (3-4 mg/day), with a flexible dosing regimen within the specified intervals during the first 6 weeks based on tolerability and clinical improvement. During the second half of treatment period 1, patients were maintained on a fixed dose at the final dose achieved during the initial 6-week flexible dosing phase. The study drug was administered twice daily (in equal doses) and was blinded using a double-blinding method. | NR |
| **Pande et al. 2003** | The 1-week, single-blind placebo lead-in phase was intended to establish the stability of generalized anxiety disorder symptoms and eliminate the effects of prior treatments. Study medication was titrated during the first 6 days. On day 1, subjects received one-sixth of the randomly assigned dose, which was then increased daily until the targeted dose was reached. Following 4 weeks of treatment, the final efficacy assessments were made (termination visit). Study medication dose was tapered over 1 week, and the follow-up visit was conducted. | Patients were required to be free of psychotropic medications for 2 weeks (5 weeks for fluoxetine). No psychotropic medications were allowed during the study with the exception of zolpidem (5 mg), which was permitted on an as-needed basis for extreme sleeplessness. |
| **Cvjetkovic-Bosnjak et al. 2015** | NR | NR |
| **Kasper et al. 2009** | Pregabalin treatment was started at a dose of 150 mg twice daily for the first week; thereafter, PGB dosing was flexible, based on clinical response and tolerability, in the range of 300–600 mg/day, administered twice daily. Venlafaxine treatment was started at a dose of 75 mg/ day (administered in the morning, with matching placebo in the evening) for the first week. Thereafter, velnafaxine dosing was flexible, in the range of 75–225 mg/day, administered in the morning (with matching placebo in the evening). | 1-week drug-free |
| **Montgomery et al. 2006** | Following a 1-week screening period, patients were randomly assigned to 1 of 4 treatment groups for 6 weeks of double-blind treatment, all administered on a twice-per-day (b.i.d.) dosing schedule: pregabalin 400 mg/day, pregabalin 600 mg/day, venlafaxine 75 mg/day, or matched placebo. Patients assigned to pregabalin 400 mg/day received 100 mg/day for 2 days, then 200 mg/day for 2 days, before receiving the full dosage of 400 mg/day on day 5. Patients assigned to pregabalin 600 mg/day received 150 mg/day for 2 days, 300 mg/day for 2 days, and 450 mg/day for 2 days, before receiving the full dosage of 600 mg/day after their day 7 visit. Patients assigned to venlafaxine began treatment at the full 37.5 mg b.i.d. dosage. Six weeks of double-blind treatment were followed by a 1-week, double-blind taper and follow-up phase. | NR |
| **Silva Miguel et al. 2013** | NR | NR |
| **Vera-Llonch et al. 2010** | NR | NR |
| **Rickels et al. 2012** | Treatment with pregabalin was initiated at 75 mg twice a day (BID) for the first week. At each weekly visit during the first 6 weeks of the double-blind treatment phase, the dose of pregabalin was increased by 75 mg BID if the patient continued to have an HAM-A total score of at least 8 and they were tolerating the current dose level. The maximum permissible dose of pregabalin was 300mg BID. Dose escalation was not permitted after week 6. | NR |
| **Feltner et al. 2008** | The treatment was initiated at 100 mg TID, with the 450 and 600 mg dosage groups increasing to 450 mg per day on day 4, and the highest dosage group increasing on day 7 to 600 mg per day. | NR |
| **Hadley et al. 2012** | Started dose of 75 mg b.i.d. and then titrated over2 weeks to 150 mg b.i.d. pregabalin. After that, pregabalin could be titrated, based on tolerability and efficacy, in increments of 150 mg/week, in the dosing range of 150–600 mg/day. At the same double-blind baseline visit, patients were instructed to begin tapering off their alprazolam at a dose reduction rate of 25% per week. Patients were permitted up to 6 weeks to complete the alprazolam taper. Benzodiazepine free patients were then continued in double-blind treatment with pregabalin or placebo for the remaining 6 weeks, at which point they were tapered off study medication over a 1-week period. During the taper phase, patients were provided with a ‘rescue medication’ packet consisting of the current dose of alprazolam, which they were instructed not to take unless they developed severe or intolerable symptoms of withdrawal or rebound anxiety. They were also required to contact their study physician before taking rescue medication. During the benzodiazepine-free phase, the rescue medication packet consisted of 1-mg tablets of alprazolam. | NR |
| **Montgomery et al. 2008** | Pregabalin treatment was initiated at 50 mg/day, followed by an increase to 100 mg/day on day 3, and 150 mg/day on day 5. Dosing was flexible from weeks 1 to 6 in the range of 150–600 mg/day, administered either twice daily or three times daily. Patients were maintained on the same dose of medication from weeks 6 to 8. | 1-week drug-free screening period |

**Supplementary Table 1.** Treatment schemes and free-drug period of the included studies.

NR: Not reported.

**References**

Rickels, K., Pollack, M. H., Feltner, D. E., Lydiard, R. B., Zimbroff, D. L., Bielski, R. J., Tobias, K., Brock, J. D., Zornberg, G. L., & Pande, A. C. (2005). Pregabalin for treatment of generalized anxiety disorder: a 4-week, multicenter, double-blind, placebo-controlled trial of pregabalin and alprazolam. *Archives of general psychiatry*, 62(9), 1022–1030. https://doi.org/10.1001/archpsyc.62.9.1022.

Feltner, D. E., Crockatt, J. G., Dubovsky, S. J., Cohn, C. K., Shrivastava, R. K., Targum, S. D., Liu-Dumaw, M., Carter, C. M., & Pande, A. C. (2003). A randomized, double-blind, placebo-controlled, fixed-dose, multicenter study of pregabalin in patients with generalized anxiety disorder. *Journal of clinical psychopharmacology*, 23(3), 240–249. https://doi.org/10.1097/01.jcp.0000084032.22282.ff.

Kasper, S., Iglesias-García, C., Schweizer, E., Wilson, J., DuBrava, S., Prieto, R., Pitman, V. W., & Knapp, L. (2014). Pregabalin long-term treatment and assessment of discontinuation in patients with generalized anxiety disorder. *The international journal of neuropsychopharmacology*, 17(5), 685–695. https://doi.org/10.1017/S1461145713001557.

Pande, A. C., Crockatt, J. G., Feltner, D. E., Janney, C. A., Smith, W. T., Weisler, R., Londborg, P. D., Bielski, R. J., Zimbroff, D. L., Davidson, J. R., & Liu-Dumaw, M. (2003). Pregabalin in generalized anxiety disorder: a placebo-controlled trial. *The American journal of psychiatry*, 160(3), 533–540. https://doi.org/10.1176/appi.ajp.160.3.533.

Cvjetkovic-Bosnjak, M., Soldatovic-Stajic, B., Babovic, S. S., Boskovic, K., & Jovicevic, M. (2015). Pregabalin versus sertraline in generalized anxiety disorder. An open label study. *European review for medical and pharmacological sciences*, 19(11), 2120–2124.

Kasper, S., Herman, B., Nivoli, G., Van Ameringen, M., Petralia, A., Mandel, F. S., Baldinetti, F., & Bandelow, B. (2009). Efficacy of pregabalin and venlafaxine-XR in generalized anxiety disorder: results of a double-blind, placebo-controlled 8-week trial. *International clinical psychopharmacology*, 24(2), 87–96. https://doi.org/10.1097/yic.0b013e32831d7980.

Silva Miguel, L., Silva Miguel, N., & Inês, M. (2013). A cost-utility analysis of pregabalin versus venlafaxine XR in the treatment of generalized anxiety disorder in Portugal. *Cost effectiveness and resource allocation* : C/E, 11(1), 8. https://doi.org/10.1186/1478-7547-11-8.

Vera-Llonch, M., Dukes, E., Rejas, J., Sofrygin, O., Mychaskiw, M., & Oster, G. (2010). Cost-effectiveness of pregabalin versus venlafaxine in the treatment of generalized anxiety disorder: findings from a Spanish perspective. *The European journal of health economics* : HEPAC : health economics in prevention and care, 11(1), 35–44. https://doi.org/10.1007/s10198-009-0160-7.

Rickels, K., Shiovitz, T. M., Ramey, T. S., Weaver, J. J., Knapp, L. E., & Miceli, J. J. (2012). Adjunctive therapy with pregabalin in generalized anxiety disorder patients with partial response to SSRI or SNRI treatment. *International clinical psychopharmacology*, 27(3), 142–150. https://doi.org/10.1097/YIC.0b013e328350b133.

Feltner, D., Wittchen, H. U., Kavoussi, R., Brock, J., Baldinetti, F., & Pande, A. C. (2008). Long-term efficacy of pregabalin in generalized anxiety disorder. *International clinical psychopharmacology*, 23(1), 18–28. https://doi.org/10.1097/YIC.0b013e3282f0f0d7.

Hadley, S. J., Mandel, F. S., & Schweizer, E. (2012). Switching from long-term benzodiazepine therapy to pregabalin in patients with generalized anxiety disorder: a double-blind, placebo-controlled trial. *Journal of psychopharmacology* (Oxford, England), 26(4), 461–470. https://doi.org/10.1177/0269881111405360.

Montgomery, S., Chatamra, K., Pauer, L., Whalen, E., & Baldinetti, F. (2008). Efficacy and safety of pregabalin in elderly people with generalised anxiety disorder. *The British journal of psychiatry : the journal of mental science*, 193(5), 389–394. https://doi.org/10.1192/bjp.bp.107.037788.
