# Supplementary material for: Does pregabalin offer potential as a first-line therapy for generalized anxiety disorder? A meta-analysis of efficacy, safety, and cost-effectiveness
Source: Front Pharmacol. 2025 Feb 7;16:1483770. doi: 10.3389/fphar.2025.1483770 (PMC11842937; doi:10.3389/fphar.2025.1483770)
Supplement: Supplementary file 3 [file Supplementaryfile2.docx]

**Supplementary File 2.** Risk of bias judgement.

**ADAN study**

1. Not well reported.
2. Not reported.
3. Not reported.
4. Not reported.
5. Not reported.
6. All kind of outcomes with complete data.

**Cvjetkovic-Bosnjak et al. 2015**

1. Each patient was randomly assigned to 4 weeks of treatment with pregabalin (n=47) or sertraline (n=60).
2. Not reported.
3. Double-blind.
4. Not reported.
5. Not reported.
6. All kind of outcomes with complete data.

**Feltner et al. 2003**

1. If patients still met the study inclusion criteria at the end of the lead-in, they were randomized to receive one of the four treatments for a 4-week double-blind titration and treatment phase.
2. Not reported.
3. Double-blind.
4. Not reported.
5. Not reported.
6. All kind of outcomes with complete data.

**Feltner et al. 2008**

1. Not well defined.
2. Figure 1.
3. Double-blind.
4. Not reported.
5. Figure 1.
6. All kind of outcomes with complete data.

**Hadley et al. 2012**

1. Patients who continued to meet eligibility criteria were randomized on a one-to-one basis to 12 weeks of double blindtreatment with either pregabalin or placebo.
2. Figure 1.
3. Double-blind.
4. Not reported.
5. Figure 1.
6. All kind of outcomes with complete data.

**Kasper et al. 2009**

1. Patients were randomized to one of the three treatment groups based on a computer-generated randomization list.
2. Figure 1.
3. Double-blind.
4. Not reported.
5. Figure 1.
6. All kind of outcomes with complete data.

**Kasper et al. 2014**

1. Eligible patients were randomly assigned at baseline to a sequence of treatments.
2. Figure 2.
3. Double-blind.
4. Not reported.
5. Figure 2.
6. All kind of outcomes with complete data.

**Montgomery et al. 2006**

1. Not well defined.
2. Figure 1.
3. Double-blind.
4. Double-blind.
5. Figure 1.
6. All kind of outcomes with complete data.

**Montgomery et al. 2008**

1. This was a randomised (2:1 pregabalin:placebo).
2. Figure 1.
3. Double-blind.
4. Not reported.
5. Figure 1.
6. All kind of outcoutcomes with complete data.

**Pande et al. 2003**

1. If patients still met study inclusion criteria at the end of the lead-in phase, as confirmed by a second clinical interview with the psychiatrist, they were randomly assigned to one of the four treatment conditions.
2. Not reported.
3. Double-blind.
4. Not reported.
5. Not reported.
6. All kind of outcomes with complete data.

**Rickels et al. 2005**

1. Patients who met the study enrollment criteria completed a 1-week drug-free screening period, during which no placebo was administered and prohibited medications were washed out; then, patients were randomized, in blocks of 10, to 4 weeks of double-blind study treatment.
2. Figure 1.
3. Double-blind.
4. Not reported.
5. Figure 1.
6. All kind of outcomes with complete data.

**Rickels et al. 2012**

1. Not well defined.
2. Figure 1.
3. Double-blind.
4. Not reported.
5. Figure 1.
6. All kind of outcomes with complete data.

**Silva Miguel et al. 2013**

1. Not well defined.
2. Figure 1.
3. Double-blind.
4. Not reported.
5. Not reported.
6. All kind of outcomes with complete data.

**Vera-Llonch et al. 2010**

1. Not well defined.
2. Figure 1.
3. Double-blind.
4. Not reported.
5. Not reported.
6. All kind of outcomes with complete data.
